# Involvement of Coenzyme Q10 in Various Neurodegenerative and Psychiatric Diseases

**DOI:** 10.1155/2023/5510874

**Published:** 2023-11-01

**Authors:** Alireza Ebrahimi, Amirhossein Kamyab, Sahar Hosseini, Sedigheh Ebrahimi, Soheil Ashkani-Esfahani

**Affiliations:** ^1^Student Research Committee, Shiraz University of Medical Sciences, Shiraz, Iran; ^2^Faculty of Medicine, Fasa University of Medical Sciences, Fasa, Iran; ^3^Department of Medical Ethics, Shiraz University of Medical Sciences, Shiraz, Iran

## Abstract

Coenzyme Q10 (CoQ10), commonly known as ubiquinone, is a vitamin-like component generated in mitochondrial inner membranes. This molecule is detected broadly in different parts of the human body in various quantities. This molecule can be absorbed by the digestive system from various nutritional sources as supplements. CoQ10 exists in three states: in a of reduced form (ubiquinol), in a semiquinone radical form, and in oxidized ubiquinone form in different organs of the body, playing a crucial role in electron transportation and contributing to energy metabolism and oxygen utilization, especially in the musculoskeletal and nervous systems. Since the early 1980s, research about CoQ10 has become the interest for two reasons. First, CoQ10 deficiency has been found to have a link with cardiovascular, neurologic, and cancer disorders. Second, this molecule has an antioxidant and free-radical scavenger nature. Since then, several investigations have indicated that the drug may benefit patients with cardiovascular, neuromuscular, and neurodegenerative illnesses. CoQ10 may protect the neurological system from degeneration and degradation due to its antioxidant and energy-regulating activity in mitochondria. This agent has shown its efficacy in preventing and treating neurological diseases such as migraine, Parkinson's disease, Alzheimer's disease, Huntington's disease, amyotrophic lateral sclerosis, and Friedreich's ataxia. This study reviews the literature to highlight this agent's potential therapeutic effects in the mentioned neurological disorders.

## 1. Background

Coenzyme Q10 (CoQ10), also known as ubiquinone, is a vitamin-like component that is principally synthesized in the inner membranes of mitochondria [1]. This molecule is broadly detected in different human tissues and cells, such as plasma, erythrocytes, platelets, skeletal muscles, and breast milk with different concentrations, as well as in organelles such as mitochondria, lysosomes, the Golgi, and almost all plasma membranes [2, 3]. CoQ10 could also be found in foods such as beef, poultry, broccoli, soya oil, fish oils, peanuts, sardines, and mackerel and can be absorbed by the digestive system as a supplement.

**Figure 1:**
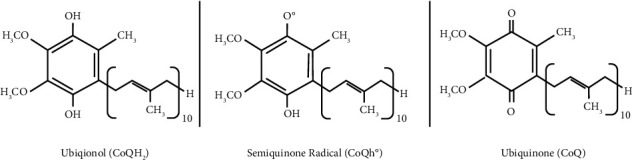
Coenzyme Q10 can exist in 3 states of oxidation: the fully oxidized ubiquinone form (CoQ), the radical semiquinone intermediate (CoQH°), and the fully reduced ubiquinol form (CoQH_2_).

CoQ10 has a lipophilic redox state and is involved in a variety of vital cellular functions, from regulating transition pores to modifying electron carrier molecules to synthesizing pyrimidine nucleotides [4]. CoQ10 can be found in three states: a reduced form (ubiquinol), in semiquinone radical form, and in an oxidized form (ubiquinone) in different organs of the human body (Figure 1), playing a crucial role in electron transportation and contributing to energy metabolism and oxygen utilization, particularly in the musculoskeletal and nervous systems [5]. At the top of these effects, CoQ10 plays a significant role in different body systems as an antioxidant and fatty acid ß-oxidation modulator [6].

Since the early 1980s, the number of studies on CoQ10 has increased because of two main reasons. First, it was observed that there is a correlation between the deficiency of this agent and disorders such as cardiovascular diseases, neurologic disorders, and cancer [7]. Second, the antioxidant and free-radical scavenger characteristics of this molecule became evident to the researchers [7]. Since then, multiple studies have shown that the agent is likely to have beneficial effects on several diseases, such as cardiovascular, neuromuscular, and neurodegenerative diseases [8, 9]. Clinical trials showed this agent could be advantageous in improving fatigue and ameliorating mitochondrial diseases [10, 11]. Furthermore, due to its antioxidative nature, boosting cellular mitochondrial function and energy metabolism, and anti-inflammatory performance, this agent has been effective in increasing the quantity and quality of oocytes and sperms and improving the reproductive function [12, 13].

Previous studies reported that CoQ10 supplementation can lead to an increment in its levels in the mitochondria of the brain, heart, skeletal muscles, liver, and kidneys of rats [14]. Moreover, according to previously published papers, supplementary CoQ10 raised the levels of CoQ10 in the human plasma and cells such as platelets and white blood cells [2, 15]. In prior investigations, the administration of solubilized ubiquinol and divided dosages of CoQ10 could result in higher bioavailability [16]. Safe dosage supplementation is considered to be up to 1200 mg/day [17, 18].

It is worth mentioning that some medications can affect CoQ10 levels in the body. Previous studies noted that statins can decrease CoQ10 production by blocking cholesterol production, which is known as an important pathway for the production of CoQ10 [19]. In other words, by targeting the mevalonate pathway in blocking cholesterol production, statins can inhibit the intracellular biosynthesis of CoQ10 [20].

**Figure 2:**
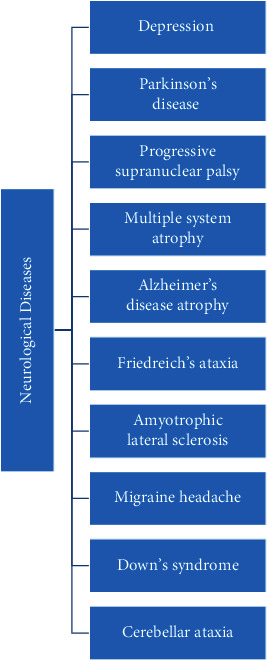
The neurological diseases that might be benefited from CoQ10 supplementation.

A growing body of evidence suggests that CoQ10 might protect the nervous system from degeneration and deterioration, mainly due to its antioxidant and energy-regulating role in mitochondria [9, 21]. In this regard, previous investigations reported the promising effects of this agent on the prevention and treatment of several neurological diseases such as migraine, Parkinson's disease, Alzheimer's disease, Huntington's disease, amyotrophic lateral sclerosis, and Friedreich's ataxia (Table 1, Figure 2) [9, 60]. The present study aimed to comprehensively review the available literature in order to highlight the possible therapeutic effects of this agent in neurological and psychiatric diseases.

## 2. Depression

Depression has been recognized as a common, debilitating neuropsychiatric disorder and an important health problem in the last century [61]. Although its prevalence may differ according to country and region, depression is still one of the major factors resulting in increasing years lost due to disability globally [62]. Conclusively, it is crucial to develop effective treatment strategies for this disorder in order to reduce its burden on patients and the healthcare system.

A high number of studies have highlighted the role of oxidative stress and immunoinflammatory responses in the pathobiology of the disease, along with the decreased levels of vitamin E, zinc, and CoQ10 [22, 61, 63, 64]. In an animal study conducted by Andalib et al., depression was induced in adult mice by intracerebroventricular infusion of a single dose of streptozotocin (0.2 mg/mouse). Then, CoQ10 was administered at a dosage of 10 mg/kg twice weekly for four weeks. The results revealed that the 4-week administration of CoQ10 could effectively ameliorate depressive-like behaviors and bioenergetic effects of streptozotocin in depressive-like animal models [65].

More than half of depressed patients who were involved in a study conducted by Maes et al. had lowered plasma CoQ10 concentrations; patients with treatment-resistant depression also had lower plasma CoQ10 levels compared to the healthy control [23]. In addition, older adults having bipolar disorder who received high doses of CoQ10 supplements showed a significant improvement in their depression symptoms severity [66].

## 3. Parkinson's Disease

Parkinson's disease (PD) is a neurodegenerative disease that is diagnosed by a progressive loss of dopaminergic neurons in the substantia nigra pars compacta, causing a loss of motor function, cognitive decline, and nonmotor symptoms [67]. PD had affected roughly six million individuals in the world by the year 2005, and its prevalence is estimated to be more than twice that by 2030 [68].

The prevalence of CoQ10 deficiency was found to be higher in PD patients [69]. According to previous studies, CoQ10 levels in platelets were depressed in patients with PD [70]. Possibly, CoQ10 serves as an antiplatelet aggregation, and the lack of CoQ10 in platelets can put patients at greater risk for stroke and cardiovascular diseases but it can also serve as an important biomarker for PD. Furthermore, the function of many PD genes has been found to be related to oxidative stress or mitochondrial function, supporting the essential role of mitochondrial involvement in neurodegenerative diseases [71].

Supplemental CoQ10 in animal models of PD reduced 1-methyl-4-phenyl-1,2,3,6-tetrahydropyridine (MPTP) levels, which is a neurotoxin involved in the emergence of the disease [24–26]. Some literature studies suggest that another substance that might play a role in the pathology of PD is iron. CoQ10 was also found to be a neuroprotective agent during iron-induced stress in dopaminergic neurons [27]. CoQ10 treatment can improve mitochondrial defects in PD patients, thus slowing the progressive decline of motor functions [28–30].

A meta-analysis conducted by Liu et al. showed that the administration of CoQ10 at a dose of 1200 mg/day was well tolerated by patients with PD. Increases in activities of daily living (ADL) were observed for CoQ10 at 1200 mg/day for 16 months compared to placebo therapy. However, the effect of CoQ10 was unclear for other components of the Unified Parkinson's Disease Rating Scale (UPDRS) [72].

However, new studies, including a phase III clinical trial, showed controversial pieces of evidence regarding the beneficial effects of CoQ10 for the treatment of PD [73, 74]. Another meta-analysis revealed that CoQ10 was well tolerated and safe in patients with PD and was not superior to placebo in terms of motor symptoms [75].

## 4. Progressive Supranuclear Palsy

Progressive supranuclear palsy (PSP) is clinically defined by postural instability and mild dementia; many patients who suffer from PSP could initially be labeled with Parkinson's disease [76]. The clinical effects of CoQ10 on PSP have been previously investigated, as the disease is caused by mitochondrial energy metabolism impairment [31].

A phase II clinical trial study suggested that CoQ10 treatment in PSP patients can improve cerebral energy metabolism and also clinical symptoms [32]. In a more recent study, sixty-one participants were treated with CoQ10 at a dosage of 2400 mg/day and with placebo for 12 months. It was revealed that taking daily dosages of CoQ10 was tolerable and safe. In addition, the patients who received CoQ10 showed a clinical decrease in total Progressive Supranuclear Palsy Rating Scale (PSPRS) scores. However, this difference was not significant statistically. In other clinical assessments, no statistically significant differences were noticeable between the test and placebo groups [77]. Perhaps irreversible damage of motor neurons specifically in areas of the brain such as the cerebellum and the primary motor cortex is responsible for such motor functions.

## 5. Multiple System Atrophy

Multiple system atrophy (MSA), formerly called Shy-Drager syndrome, is a progressive neurodegenerative disease that is characterized by a clinical triad of Parkinsonism, cerebellar ataxia, and autonomic failure. In addition, pyramidal dysfunction can also be considered as another manifestation of this syndrome [36, 78].

Due to its significance in mitochondrial function and oxidative stress, CoQ10 may be beneficial as a potential blood-based diagnostic biomarker in patients with MSA [36]. Besides, no definite treatment has been proposed for MSA till now, and physicians comply with symptomatic therapy [78]. As a result, the therapeutic effects of CoQ10 could be considered because of the mentioned roles.

A recent study demonstrated that the CoQ10 concentration was significantly decreased in the cerebellum of MSA subjects in comparison with a healthy control and other neurodegenerative diseases [33]. Also, several studies confirmed that the CoQ2 gene, encoding parahydroxybenzoate-polyprenyl transferase, which is a participant enzyme in the biosynthesis of CoQ10, was impaired in MSA cases [34, 35]. Kasai et al. also supported the idea of using CoQ10 as a treatment for this condition since they found serum CoQ10 concentrations to be lower in MSA cases compared to the control group [36].

Furthermore, a study conducted by Du et al. showed that decreased CoQ10 plasma levels were associated with the severity of motor symptoms in patients who suffer from MSA-predominant cerebellar ataxia (MSA-C) [79].

## 6. Alzheimer's Disease

Alzheimer's disease (AD) is considered as the leading cause of dementia. It is estimated that nearly 80 million of the globe's population will have suffered from the disease by 2040 [80]. Multiple pieces of evidence approve the association between the neurodegenerative processes in AD and mitochondrial oxidative damage in the brain, which could support the potential effects of CoQ10 in the treatment of the disease [37]. Supplemental CoQ10 in mouse models protected the brain from amyloid precursor protein-carboxyl terminal fragments (APP-CTFs) neurotoxicity, reduced beta-amyloid production, and also suppressed oxidative stress [37, 38]. A former previous study suggested that CoQ10 can serve as an effective treatment for AD due to its amyloidogenic effects [81].

In an in vivo study, CoQ10 treatment on a transgenic mouse model of AD showed reduced oxidative stress and amyloid pathology and improved behavioral performance in the mice. CoQ10 treatment decreased brain levels of protein carbonyls, which is a marker of oxidative stress [82]. Moreover, another study in transgenic mice showed the neuroprotective effects of CoQ10 supplementation by decreasing brain atrophy [39].

In a randomized controlled study, it was shown that the topical application of CoQ10 for the short term resulted in improvement in AD-related retinal ganglion cells (RGCs), which can reflect the salvage of some RGCs that are in the reversible transitional phase [83].

## 7. Huntington's Disease

Huntington's disease (HD) is an autosomal dominant disease characterized by psychiatric problems, progressive cognitive impairment, and choreiform movements. While symptomatic treatments are available for HD, there is no treatment to slow the progression or delay its onset [84–86]. Defective energetics in HD pathology is accepted by many experimental studies [87–89]. Therefore, it is suggested that CoQ10 may have beneficial effects in the treatment of patients suffering from HD since it improves mitochondrial function and acts as an antioxidant [42, 56, 90].

Several studies have proven the beneficial effects of CoQ10 supplementation in the treatment of animal models of HD, as it has improved behavioral and neuropathological phenotypes of the models, postponed weight loss, motor deficits, brain atrophy, and Huntingtin protein aggregation and increased brain adenosine triphosphate (ATP) levels [26, 42–46]. However, a recent study on mouse HD models questioned the previously noted benefits [91]. The Huntington's study group conducted a clinical trial in the early 20s that showed a slight slowing in functional decline in patients who used CoQ10 supplementation but statistical significance was not achieved [92].

In a recent multicenter randomized, double-blind, placebo-controlled trial, 609 patients in the early stage of HD received either CoQ10 with a dosage of 2400 mg/d or placebo and were followed for 60 months. No beneficial effect of the agent was detected in this study [93].

## 8. Friedreich's Ataxia

Friedreich's ataxia (FA) is an autosomal-recessive disorder characterized by limb ataxia, dysarthria, loss of vibration and proprioceptive sense, are flexia, abnormal eye movements, pyramidal signs, and other conditions such as cardiomyopathy [94]. Unfortunately, there is no specific biochemical or clinical marker or treatment for FA [95].

The efficacy of different treatments for FA is controversial, especially regarding the effectiveness of antioxidants such as CoQ10 [95]. Serum CoQ10 levels were reported to be lower in patients with FA [96]. Supplemental idebenone (an analog of CoQ10) was studied in treating cardiac complications of FA, which was found beneficial [97–99]. Also, CoQ10 and vitamin E supplementation modified the disease progression in a proportion of subjects with FA [96].

CoQ10 treatment in 10 FA patients increased skeletal muscle ATP production as well as the cardiac phosphocreatine to the ATP ratio; however, no improvement in echocardiogram findings or neurologic evaluation was found after six months [47]. The 4-year follow-up of the same subjects showed increased fractional shortening in echocardiographic data and slowed the progression of the International Cooperative Ataxia Rating Scale (ICARS), revealing clinical improvement, although these changes were not statistically significant [48]. Considering the vital role of genetics in FA, the effects of CoQ10 seem to be limited in this condition; however, the increased metabolic functions such as skeletal muscle ATP production are noteworthy [49].

Frataxin gene (FXN), which is known to be effective in increasing the chances of FA, has been proposed to encode a protein, assisting in iron-chaperoning during red blood cell synthesis and providing iron homeostasis [100]. Finding the link between the misexpression of FXN and CoQ10 will be the key in handling this disease in the future [101].

## 9. Amyotrophic Lateral Sclerosis

Amyotrophic lateral sclerosis (ALS) is a progressive motor neurodegenerative disease caused by the cell death of both lower and upper motor neurons which eventually reach the patient's crucial breathing muscles [102, 103]. Therefore, the mortality rate is so high and the life expectancy is so short upon diagnosis that the majority of patients only survive from two to four years following the diagnosis [104]. Despite the insufficient knowledge regarding the etiology of ALS, available evidence suggest that elevated oxidative stress and inflammatory responses play important roles in the pathogenesis of the disease [105, 106]. Conclusively, using CoQ10 supplementation in patients suffering from ALS may be beneficial due to its biological mechanisms.

Transgenic mice from ALS models who received CoQ10 supplementation had an improvement in their survival rates; perhaps certain levels of CoQ10 supplementation can slow down or improve the rate of motor neuron death in the initial stages of diagnosis [50]. Also, a study demonstrated that the oxidized form of CoQ10 was increased in ALS patients [107]. However, a systematic review of aberrations in oxidative stress markers in ALS by Wang et al. showed that there was no significant difference between blood levels of CoQ10 in the case and control groups [108]. Although some evidence shows beneficial effects of CoQ10 in this area [109], a phase II trial was not approved for the implementation of phase III [110]. These controversial findings are due to the fact that we do not know how much role CoQ10 plays in motor neuron expression.

## 10. Migraine Headache

Migraine is a common cause for health and socioeconomic burdens globally [111]. The disease is characterized by episodic attacks that affect nearly 15% of the general population or approximately one billion people in the world [112]. It highly influences the life quality of the affected patients globally [113]. The main goal of treatment is to relieve symptoms and ameliorate the functional disability of affected individuals [114, 115]. Numerous factors, including the environment, diet, and genetics, are involved in the onset of migraine headache [116].

Researchers reported brain energy metabolism being abnormally altered in all types of migraine headache [117]. In addition, the antioxidative nature of CoQ10 reduces the expression of cytokines and matrix metalloproteinase-9 (MMP-9), an enzyme involved in inflammation of nerves that could exacerbate migraine attacks [118, 119]. Therefore, treatment with CoQ10 could be helpful.

Investigations on the efficacy of CoQ10 in migraine headache showed that more than 60% of patients who received this agent had a significant decrease in the number of attacks by the end of three months; besides, a reduction in nausea was observed among CoQ10-treated patients [51–53].

The Canadian Headache Society guideline for migraine prophylaxis graded CoQ10 as a strong recommendation according to the principles of the Grading of Recommendations Assessment [120]. Also, CoQ10 therapy with dietary supplements is seen to be effective in less severe pediatric migraine patients as a prophylactic agent to avoid side effects of medications or as part of multidisciplinary treatment [54]. In addition, a meta-analysis by Zeng et al. about the efficacy of CoQ10 as a supplement for migraine showed that CoQ10 decreased migraine duration and migraine days per month compared with placebo therapy. However, the frequency and severity of attacks were not altered [111]. Yet, considering the major role of nutrition in migraine, a more controlled long-term study involving CoQ10 and other dietary supplements is needed.

## 11. Down's Syndrome

Trisomy 21, also known as Down's syndrome (DS), is the most frequent chromosomal abnormality, which characteristically accompanies neurological deficiencies and cognitive disabilities [121]. Mitochondrial dysfunction and reactive oxygen species imbalance have been observed in the cells of both animal models and in human cells, highlighting the association between oxidative imbalance and clinical manifestations of DS [122–124]. Thus, an antioxidant approach could be a promising way to delay cognitive impairment [122].

In a study by Zaki et al., it was shown that all DS patients had decreased levels of CoQ10, a potent endogenous antioxidant, in plasma [121]. In another study, the oxidized CoQ10 levels were significantly higher in patients with DS compared to the healthy group [55]. Although CoQ10 levels in both plasma and platelets elevated after supplementation, no significant DNA damage was seen in DS patients after 20 months of the therapy [125].

## 12. Cerebellar Ataxia

Cerebellar ataxias consist of a group of gait disorders that result from cerebellum dysfunction [126]. This type of ataxia is a motor disturbance that involves both planning and execution, or accuracy and coordination of movements, as a result of wide-ranging pathologies involving the cerebellum [127]. CoQ10 is a key mitochondrial respiratory chain cofactor, and its primary deficiency could lead to cerebellar ataxia [128]. So, it could be proposed as a potential therapy for cerebellar ataxia.

Several pieces of evidence support the correlation between CoQ10 deficiency measured in fibroblasts and/or muscles and cerebellar ataxia, specifically in familial cerebellar ataxia [56–59]. Patients with cerebellar ataxia and CoQ10 deficiency showed cerebellar atrophy on magnetic resonance imaging (MRI) [129]. CoQ10 supplementation was found to be effective for improving symptoms of cerebellar ataxia in CoQ10-deficient patients [56, 130–132]. In a study by Malicdan et al., it was shown that after three months of treatment with CoQ10 with a dosage of 15 mg/kg/d, patients' ataxia scores reduced significantly [6].

## 13. Conclusion

As CoQ10 is an essential substance for the optimal function of all cell types due to its role in the electron transport chain for cell energy regulation, and it is expected that its usage as a supplement will depict favorable responses in different diseases, especially those related to aging, including neurodegenerative diseases such as Parkinson's, Alzheimer's, and depression. Based on these investigations, CoQ10 may be suggested as a supplement for all elderly people because of its supportive role in age-related issues. However, further support for clinical investigations is required to determine the roles and impacts of CoQ10 in different conditions, and we are at the beginning of this long journey.

## Figures and Tables

**Table 1:** A review of CoQ10 effects on neurological diseases.

Neurological diseases	Depression	(1) Correlation with CoQ10 deficiency has been reported	[22, 23]
	Parkinson's disease	(1) Supplemental CoQ10 improved 1-methyl-4-phenyl-1,2,3,6-tetrahydropyridine (MPTP)	[24–30]
		(2) Neuroprotective agent during iron-induced stress in dopaminergic neurons	
		(3) Improving mitochondrial defect; therefore, slowing the progressive decline of function	
	Progressive supranuclear palsy	(1) Improve mitochondrial energy metabolism	[31, 32]
		(2) Improve cerebral energy metabolism and also clinical problems	
	Multiple system atrophy (Shy-DragerSx)	(1) Correlation with CoQ10 deficiency has been reported	[33–36]
	Alzheimer's disease	(1) Protection of the brain from APP-CTF neurotoxicity reduced beta-amyloid 42 productions and also suppressed oxidative stress	[37–39, 39–41]
		(2) Reversing intracerebroventricular-streptozotocin (ICV-STZ) effects in the hippocampus	
		(3) Protective effect against brain atrophy	
	Huntington's disease	(1) Increment of brain ATP levels and improvement in symptoms	[26, 42–46]
	Friedreich's ataxia	(1) Increased skeletal muscle ATP production and cardiac phosphocreatine to ATP ratio	[47–49]
		(2) Increased fractional shortening in echocardiographic data and slowing the progression of the International Cooperative Ataxia Rating Scale (ICARS)	
	Amyotrophic lateral sclerosis	(1) Improvement in survival	[50]
	Migraine headache	(1) The decrease in the number of days with migraine headache	[51–54]
		(2) Reduction in nausea and frequency	
		(3) Effective in pediatric migraines as a prophylactic agent	
	Down's syndrome	(1) Correlation with CoQ10 has been reported	[55]
	Cerebellar ataxia	(1) Correlation with CoQ10 deficiency has been reported	[56–59]

## Data Availability

The data used to support the findings of this study are available upon reasonable request from the corresponding author.
